# Host and Bacterial Proteins That Repress Recruitment of LC3 to *Shigella* Early during Infection

**DOI:** 10.1371/journal.pone.0094653

**Published:** 2014-04-10

**Authors:** Leigh A. Baxt, Marcia B. Goldberg

**Affiliations:** 1 Department of Medicine, Massachusetts General Hospital, Cambridge, Massachusetts, United States of America; 2 Department of Microbiology and Immunobiology, Harvard Medical School, Boston, Massachusetts, United States of America; Indian Institute of Science, India

## Abstract

*Shigella* spp. are intracytosolic gram-negative pathogens that cause disease by invasion and spread through the colonic mucosa, utilizing host cytoskeletal components to form propulsive actin tails. We have previously identified the host factor Toca-1 as being recruited to intracellular *S. flexneri* and being required for efficient bacterial actin tail formation. We show that at early times during infection (40 min.), the type three-secreted effector protein IcsB recruits Toca-1 to intracellular bacteria and that recruitment of Toca-1 is associated with repression of recruitment of LC3, as well as with repression of recruitment of the autophagy marker NDP52, around these intracellular bacteria. LC3 is best characterized as a marker of autophagosomes, but also marks phagosomal membranes in the process LC3-associated phagocytosis. IcsB has previously been demonstrated to be required for *S. flexneri* evasion of autophagy at late times during infection (4–6 hr) by inhibiting binding of the autophagy protein Atg5 to the *Shigella* surface protein IcsA (VirG). Our results suggest that IcsB and Toca-1 modulation of LC3 recruitment restricts LC3-associated phagocytosis and/or LC3 recruitment to vacuolar membrane remnants. Together with published results, our findings suggest that IcsB inhibits innate immune responses in two distinct ways, first, by inhibiting LC3-associated phagocytosis and/or LC3 recruitment to vacuolar membrane remnants early during infection, and second, by inhibiting autophagy late during infection.

## Introduction

*Shigella* spp. are intracytoplasmic bacterial pathogens that cause diarrheal disease by invasion and spread through the colonic epithelium [1]–[3]. *Shigella* entry into cells is mediated by a type three secretion system, a needle-like apparatus that injects effector proteins directly into the host cell cytoplasm to modulate host cell functions. Contact with host cells triggers delivery of early effector proteins, which induce rearrangements of the actin cytoskeleton resulting in membrane ruffling and bacterial uptake, which is followed shortly thereafter by bacterial escape from the uptake vacuole [4], [5]. Within the host cytoplasm, *Shigella* triggers the polymerization of a propulsive tail that consists of polymerized host actin and leads to dissemination of *Shigella* in the intestinal epithelium [6].

The *Shigella* outer membrane protein IcsA, the host actin nucleation-promoting factor N-WASP, and Toca-1 are required for efficient actin tail formation [7]–[11]. In resting cells, N-WASP is maintained in an auto-inhibited state [6], [12], [13]. In the context of *Shigella* actin tail formation, Toca-1 activates N-WASP by relieving N-WASP auto-inhibition [11]. Toca-1 is recruited to bacteria in a manner that depends on type three secretion [11], although the effector protein responsible for Toca-1 recruitment has been unknown.

We sought to characterize the mechanism and function of Toca-1 recruitment to intracellular *Shigella*. We identified IcsB as being required for recruitment of Toca-1. We found that early during infection, IcsB and Toca-1 repress recruitment of LC3 to the vicinity of intracellular *S. flexneri*. LC3 is best characterized as a marker of autophagy, a bulk degradation process used by cells to eliminate from the cytosol undesirable or invasive components, including protein aggregates, damaged organelles, and intracellular pathogens [14]. Of note, late during infection, IcsB has been shown to inhibit anti-*Shigella* autophagy [15]. However, LC3 is also central to LC3-associated phagocytosis, a process that occurs early upon uptake of pathogens and other cargo, involves several components of the classical autophagy pathway, and promotes phagosome maturation [16]–[18]. We demonstrate that the function of IcsB we characterize here is distinct from its previously described role in inhibition of autophagy [15] and is most consistent with LC3-associated phagocytosis and/or LC3 recruitment to vacuolar membrane remnants. Together, these findings indicate that IcsB and the host protein Toca-1 modulate LC3 recruitment around *S. flexneri* early during infection.

## Materials and Methods

### Bacterial strains, plasmids, and growth conditions

Strains and plasmids used in this study are listed in Table 1. All *S. flexneri* strains are isogenic to 2457T, and infectivity was similar for all strains. To generate the Δ*icsB* strain, the kanamycin cassette was removed from strain 2457T Δ*icsB*::FRT-km-FRT using FLP recombinase to generate a nonpolar, unmarked isogenic *icsB* mutant [19]. Cultures of *S. flexneri* strains were started from colonies that were Congo red positive. All strains were cultured in tryptic soy or Luria broth or agar with appropriate antibiotics and with or without addition of 0.2% glucose. Bacteria were grown at 37°C with shaking. Induction of transcription of genes under the control of IPTG-inducible promoters was performed at 37°C for 1 hr by addition IPTG to 100 µM.

**Table 1:** Strains and plasmids used in this study.

Strain or Plasmid	Genotype or description	Reference
**Bacterial strains**		
Wild-type	Wild-type serotype 2a *S. flexneri* strain 2457T	[47]
Δ*icsB* (km)	2457T *icsB*::FRT-km-FRT	Gift of Cammie Lesser
Δ*icsB*	2457T *icsB*::FRT	This study
*icsA*	2457T pWR100 *icsA*::Ω	[48]
*icsA* Δ*icsB*	2457T pWR100 *icsA*::Ω *icsB*	This study
Δ*ipaB* Δ*ipaC*	2457T *ipaB ipaC*	Gift of Cammie Lesser
SL1344	*S*. Typhimurium	Lab stock
BL21 DE3	*E. coli* K-12	Lab stock
**Plasmids**		
Myc-Toca-1	pCS2+MT full length Toca-1,	[12]
Toca-1_1–477_	pCS2+MT Toca-1 truncation mutant (amino acids 1-477)	[12]
Toca-1_1–293_	pCS2+MT Toca-1 truncation mutant (amino acids 1–293)	[12]
Toca-1_105–547_	pCS2+MT Toca-1 truncation mutant (amino acids 105–547)	[12]
Toca-1_245–547_	pCS2+MT Toca-1 truncation mutant (amino acids 245–547)	[12]
Toca-1_372–547_	pCS2+MT Toca-1 truncation mutant (amino acids 372–547)	[12]
Toca-1_483–547_	pCS2+MT Toca-1 truncation mutant (amino acids 483–547)	[12]
Toca-1_105–477_	pCS2+MT Toca-1 truncation mutant (amino acids 105–477)	This study
Toca-1_245–477_	pCS2+MT Toca-1 truncation mutant (amino acids 245–477)	This study
Toca-1_105–373_	pCS2+MT Toca-1 truncation mutant (amino acids 105–373)	This study
Toca-1_245–373_	pCS2+MT Toca-1 truncation mutant (amino acids 245–373)	This study
LC3-GFP	pEGFP-C1-LC3	[49]
pDSW206-flag	IPTG inducible expression of 3xflag	Gift of Jon Beckwith
pDSW206-OspF-flag	IPTG inducible expression of OspF-3xflag	Gift of Cammie Lesser
pDSW206-IcsB-flag	IPTG inducible expression of IcsB-3xflag	Gift of Cammie Lesser

Myc-Toca-1 truncation mutants used in this study were generated by PCR amplification of the DNA fragments encoding the relevant portions of Toca-1, using Myc-Toca-1 as template and subcloning of these fragments into pCS2+MT. Sequence analysis was performed to verify that each construct was correct. The sequences of the primers used in this study are available from the authors upon request.

### Cell lines and culture conditions and transfection

Cells were cultured in high glucose DMEM (Gibco) supplemented with 10% fetal calf serum and pen/strep at 37°C with 5% CO_2_. A431 human epidermoid carcinoma cells, originally derived from a solid tumor [20], stably containing either an empty lentiviral plasmid or plasmid encoding shRNA directed against Toca-1 (gift of Andrew Craig [21]) were maintained under selection with 1 µg/ml puromycin. HeLa cells (ATCC), 293T cells (ATCC), and mouse embryonic fibroblasts (MEFs) ([22]; gift of David Sacks) were maintained under the same conditions.

On the day prior to transfections, 3×10^5^ cells were seeded onto glass coverslips. Monolayers of HeLa and A431 cells were transfected using 1.5 µg DNA and 4.5 µl Fugene 6 (Promega) transfection reagent in DMEM to a total volume of 100 µl, followed by 20 min incubation at room temperature before adding to the cells. Transfected cells were infected with bacteria 24 hr after transfection.

### Immunoprecipitation, co-precipitation, and western blot analysis

293T cells were transfected with selected plasmids using the calcium phosphate method. Forty-eight hr after transfection, cells were lysed in chilled lysis buffer (150 mM NaCl, 1 mM EDTA, 50 mM Tris pH 7.4, 1% Triton X-100, protease inhibitor cocktail (Roche)) for 30 min and cleared by centrifugation in a microfuge at 15,000 rpm for 15 min at 4°C. Secreted bacterial proteins were bound to antibody-conjugated beads by incubation of the beads with bacterial culture supernatants 1 hr at 4°C, followed by 3 washes in lysis buffer. The prepared beads were then incubated with cleared cell lysates overnight at 4°C, followed by recovery by centrifugation. Beads were then washed 6 times with lysis buffer, resuspended in SDS-PAGE sample buffer, and analyzed by western blot. Primary antibodies used for western blot analysis were anti-Myc (Clontech), anti-Flag M2 (Sigma), anti-HA (Covance), each used at 1∶1000. Secondary antibodies were HRP-conjugated True Blot antibodies (eBioscience), used at 1∶5000.

For bacterial co-precipitation experiments, proteins were synthesized in *E. coli* BL21-DE3. Overnight cultures were back diluted and grown at 16°C for several hours before inducing expression of the target proteins from IPTG-inducible and arabinose-inducible promoters with 100 µM IPTG and 0.2% arabinose overnight. The following day, bacteria were recovered by centrifugation and lysed with sonication in chilled lysis buffer (150 mM NaCl, 1 mM EDTA, 50 mM Tris pH 7.4, protease inhibitor cocktail). Lysates were centrifuged in a microfuge at 15,000 rpm for 5 min at 4°C. Soluble fractions were incubated with amylose or cobalt resin for 2 hr at 4°C. Resins with bound protein were then washed 4 times in lysis buffer, recovered by centrifugation, and resuspended in SDS-PAGE sample buffer.

### Bacterial infections of mammalian cells

Bacteria were grown in liquid media at 37°C with aeration to an absorbance 600 of 0.2–0.5, recovered by centrifugation, resuspended in cell culture media, and added to cell monolayers at a multiplicity of infection of 100–200 for *S. flexneri* and 50–100 for *S*. Typhimurium (bacteria:cell). When appropriate, 100 µM IPTG was added to the bacterial culture for the last 1 hr of growth and throughout the cell infection. To bring bacteria into close contact with the cells, infected monolayers were centrifuged for 10 min at 2,000 rpm. Infected monolayers were then incubated at 37°C for 15 min, washed once with pre-warmed cell culture media, and incubated at 37°C for an additional 15 min for *S. flexneri* or 35 min for *S*. Typhimurium. Monolayers were then fixed in 3.7% paraformaldehyde in F-buffer for 20 min and permeabilized with 1% Triton-X-100. Immunofluorescence labeling was performed using as primary antibodies, Toca-1 (gift of G. Scita, used at a dilution of 1∶20), P62 (BD Biosciences, used at a dilution of 1∶100), NDP52 (Abcam, used at a dilution of 1∶100), FK2 (Enzo Life Sciences, used at a dilution of 1∶100), and Galectin-3 (used at a dilution of 1∶100, BD) with as secondary antibodies, anti-mouse Alexa 488, anti-rabbit Alexa 488, anti-mouse Alexa 568, and anti-rabbit Alexa-568 (each used at a 1∶100 dilution, and each from Invitrogen). Staining of actin, cholesterol and DNA was performed using Alexa 568 phalloidin (Invitrogen), filipin complex (Sigma) and 5 µM DAPI (Invitrogen).

For the quantification of bacterial survival in cells (gentamicin protection assay), MEFs were seeded at 3×10^5^ cells per well in 12-well plates the day prior to infection, giving approximately 95% confluence on the day of infection. *S. flexneri* strains were grown as described above and added to cell monolayers at a multiplicity of infection of 0.0001 (bacteria:cell). The bacterial inoculum was plated for quantification. Infected monolayers were centrifuged for 10 min at 2,000 rpm and incubated at 37°C for 45 min. Cell monolayers were then washed once with pre-warmed DMEM and incubated for an additional 4 hrs at 37°C with fresh DMEM containing 25 µg/ml gentamicin, which kills extracellular but not intracellular bacteria. Infected monolayers were then washed twice with phosphate buffered saline and lysed in 1% Triton X-100 to release the intracellular bacteria. Lysates containing the bacteria were plated on Congo red agar and bacterial colonies were quantified 16 hrs later.

### Microscopy analysis

Experimental samples were blinded, infected cells were identified using the DAPI channel, and a minimum of ten cells was analyzed for each condition. For the analysis of Toca-1 and LC3 recruitment, a bacterium was counted as positive if labeling was observed anywhere on the periphery of the bacterium. For quantification of actin tail formation, bacteria were counted as having a tail if the polymerized actin adjacent to the bacterium was ≥0.83 uM in length, as described [11]. For quantification of autophagosome formation in MEFs, cells were counted as positive if at least one intracellular bacterium was surrounded by LC3-GFP.

### Data analysis

For all sets of experiments, three or more independent experiments were performed, data were collected in a blinded manner, and standard statistical tools were applied to assess for significant differences among experimental results.

## Results

### The type three-secreted effector IcsB is required for recruitment of Toca-1 around intracellular *Shigella*

**Figure 1:**
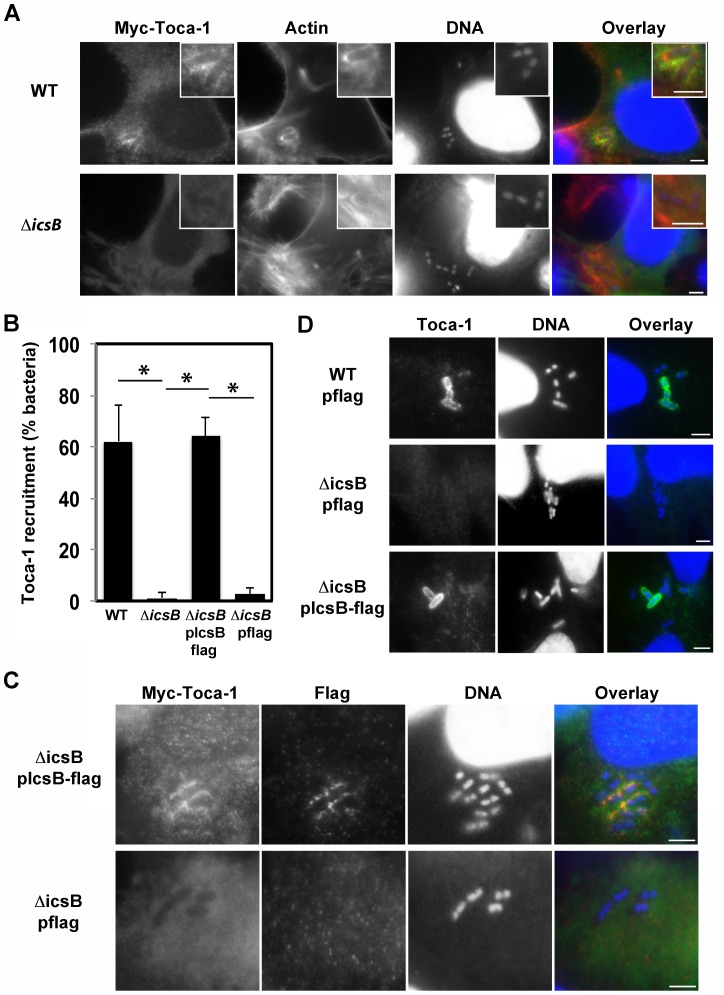
IcsB is required for recruitment of Toca-1. (A) Wild-type (WT) or Δ*icsB S. flexneri* infection (40 min.) of HeLa cells that had been transfected with Myc-Toca-1, with immunofluorescence using antibody to Myc and staining with phalloidin (polymerized actin) and DAPI (DNA). (B) Association with Myc-Toca-1 with intracellular wild-type (WT) or Δ*icsB S. flexneri* and complemented strains. Data represent the mean ± S.D. of three independent experiments. *P<0.05. (C) Infection (40 min.) by Δ*icsB S. flexneri* and IcsB-flag complemented strain of HeLa cells that had been transfected with Myc-Toca-1, with immunofluorescence using antibodies to Myc and flag. (D) Infection (40 min.) by Δ*icsB S. flexneri* and IcsB-flag complemented strain of HeLa cells, with immunofluorescence using antibody to Toca-1 and stained with DAPI. Scale bars, 5 µm.

Previous studies from our lab have shown that Toca-1 is recruited to intracellular *Shigella* in a manner that depends on type three secretion [11]. To determine which type three-secreted effector protein was required for recruitment of Toca-1 to bacteria, we screened for loss of Toca-1 recruitment in HeLa cells infected with each among a panel of 21 isogenic *S. flexneri* strains harboring single deletions of genes encoding type three effector proteins for 40 min., the time at which Toca-1 recruitment peaks (Fig. S1). A single mutant, Δ*icsB*, showed a dramatic decrease in recruitment of Myc-Toca-1 (wild-type, 62±14% versus Δ*icsB*, <1±2% of intracellular bacteria, p = 0.006, Fig. 1A and B). Expression of IcsB from a plasmid in the Δ*icsB* mutant restored Toca-1 recruitment to wild-type levels (Fig. 1B), confirming that the defect in Toca-1 recruitment observed for the deletion strain was due to the absence of IcsB. Immunolocalization revealed that like Toca-1, IcsB formed rings around intracellular bacteria (Fig. 1C) and that IcsB and Toca-1 co-localized around the same intracellular bacteria (Fig. 1C). Identical results were observed using an antibody to endogenous Toca-1 (Fig. 1D), indicating that the over-expression of transfected Myc-Toca-1 *per se* did not contribute to the recruitment phenotype. Toca-1 was detectable around bacteria within membrane ruffles (Fig. S2), suggesting that Toca-1 is recruited as extracellular bacteria are taken up into vacuoles. These data demonstrate that IcsB is required for recruitment of Toca-1 to *S. flexneri* and suggest that this occurs during bacterial entry.

### IcsB interacts with Toca-1

**Figure 2:**
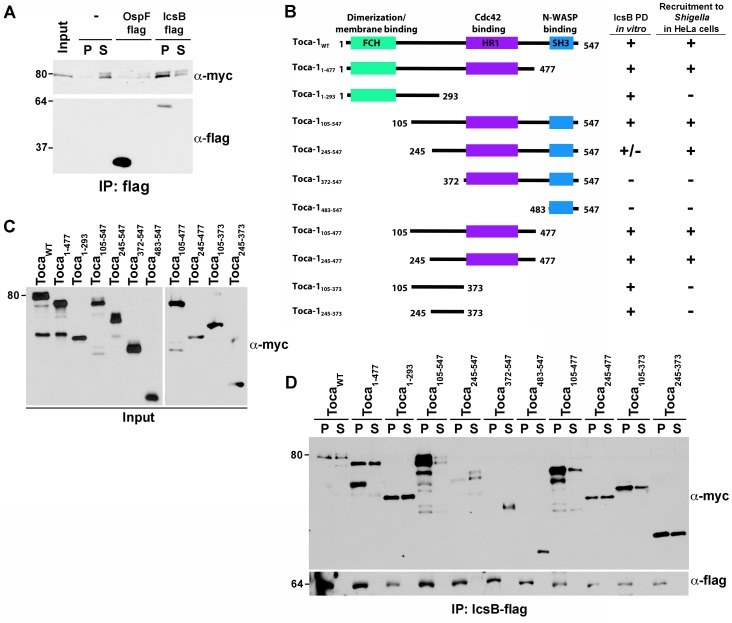
IcsB interacts with the HR1 and adjacent coiled coil region of Toca-1. (A) Immunoprecipitation of Myc-Toca-1 with IcsB-flag. Proteins secreted by *S. flexneri* strains carrying no plasmid, IcsB-flag, or OspF-flag were bound to anti-flag beads, which were then used to precipitate Myc-Toca-1 from cell lysates. Western blot analysis. Input, cell lysate. (B) Schematic of Toca-1 domain structure and truncation mutants, indicating pull down by IcsB *in vitro* and recruitment to wild type *S. flexneri* in cells. (C and D) Immunoprecipitation of Myc-Toca-1 and its truncation mutants with IcsB-flag, as in panel (A). (C) Input. Panels are from the same blot. (D) Precipitated proteins. Western blot analysis. P, pellet; S, supernatant.

The dependence of Toca-1 recruitment on IcsB and the co-localization of IcsB with Toca-1 (Fig. 1) suggested that IcsB might interact with Toca-1. To test this, IcsB-flag that had been recovered on beads from bacterial culture supernatants was used to precipitate proteins from lysates of 293T cells that had been transfected with Myc-Toca-1 (see Methods). IcsB-flag beads precipitated Myc-Toca-1 in a specific manner, since Myc-Toca-1 was not precipitated by beads that had been incubated with culture supernatant alone or with a flag-tagged derivative of the type three secreted protein OspF (Fig. 2A).

To define the region of Toca-1 that interacts with IcsB, we tested the ability of IcsB-flag to precipitate derivatives of Toca-1 in which specific domains had been deleted (Fig. 2B). The results of this analysis narrowed the interaction to a region of Toca-1 that includes the protein kinase C-related kinase homology region 1 (HR1) and a coiled coil domain that lies between the Fer/Cip4 homology (FCH) and HR1 domains (Fig. 2B–D). To determine whether the region required for *in vitro* interaction between the two proteins correlated with that required for recruitment of Toca-1 to *Shigella* in infected cells, we analyzed recruitment of each truncation mutant to intracellular *S. flexneri* in HeLa cells (Figs. 2B and S3). The recruitment of the Toca-1 truncation mutants to intracellular bacteria correlated with the precipitation experiments with three exceptions (Toca-1_105–373_, Toca-1_245–373_, and Toca_245–547_). Toca-1_105–373_ and Toca-1_245–373_ interacted by precipitation, but failed to be recruited in the context of infection; these discrepancies are likely due to non-native tertiary structure or the requirement for an additional host protein to promote localization to intracellular bacteria.

In addition, Toca_245–547_ reproducibly bound poorly in vitro, but localized well to *S. flexneri* in cells (Fig. 2D and S3). Whereas we do not understand these findings, we speculate that in this construct, the Toca-1 SH3 domain may partially interfere with binding to IcsB under the *in vitro* conditions, but not inside cells, where perhaps another interaction prevents the interference. Consistent with this possibility is that Toca-1 constructs that contain the core IcsB binding domain (residues 245–477) and lack the SH3 domain were generally precipitated better by IcsB than the corresponding constructs that contain the SH3 domain.

**Figure 3:**
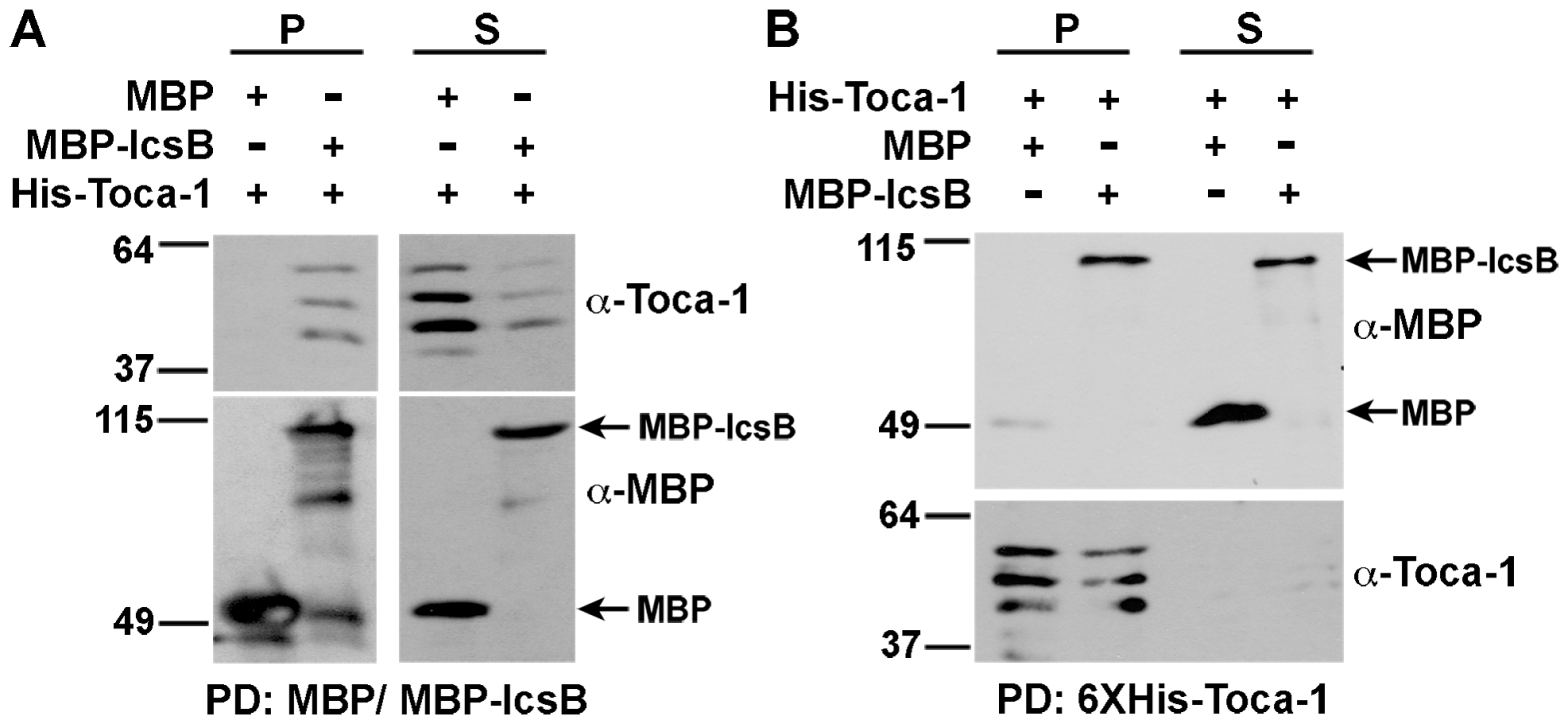
Toca-1 and IcsB reciprocally co-precipitate. Reciprocal co-precipitation of His-Toca-1_105–477_ and MBP-IcsB from soluble fractions of *E. coli* BL21 DE3 that co-express the two proteins or the relevant controls, using amylose (for MBP, panel A) or cobalt (for His, panel B) resin. Western blot analysis. The apparent degradation observed for the Toca-1 construct was independent of IcsB. Apparent molecular weights in kilodaltons are indicated to the left of the blots. P, pellet; S, supernatant.

To test whether the interaction between IcsB and Toca-1 is dependent on other *S. flexneri* or other mammalian proteins, we performed co-precipitation analysis in *E. coli*. In this system, MBP-IcsB and a truncation mutant of Toca-1 that was still recruited to intracellular *S. flexneri* (Toca-1_105–477_) reciprocally interacted by co-precipitation (Fig. 3). The interaction was specific, since when MBP was produced with Toca-1, no interaction was observed (Fig. 3). These results indicate that the interaction between IcsB and Toca-1 is independent of *S. flexneri* and other mammalian proteins.

### IcsB is associated with decreased early LC3 recruitment

Since IcsB is implicated in inhibition of autophagy at late time points (4–6 hr) during *S. flexneri* infection [15], we considered whether IcsB recruitment of Toca-1 early during infection might alter autophagy related processes at that time. Recognition of cytosolic bacteria by the autophagy pathway (anti-bacterial autophagy) may involve ubiquitination of bacterial surface proteins or signaling via pattern-recognition molecules, including Toll-like receptors (TLRs), NOD-like receptors (NLRs), and/or diacylglycerol (DAG) [23], . Specificity is provided by receptors that function as adaptor proteins, referred to as sequestrosome 1/P62-like receptors (SLRs) [25], including NDP52 and P62 [26]. These receptor/adaptor proteins recognize ubiquitin and other markers of cargo and in turn activate recruitment of the lipidated form of Atg8, LC3 II (LC3), a canonical marker of autophagosomal membranes.

**Figure 4:**
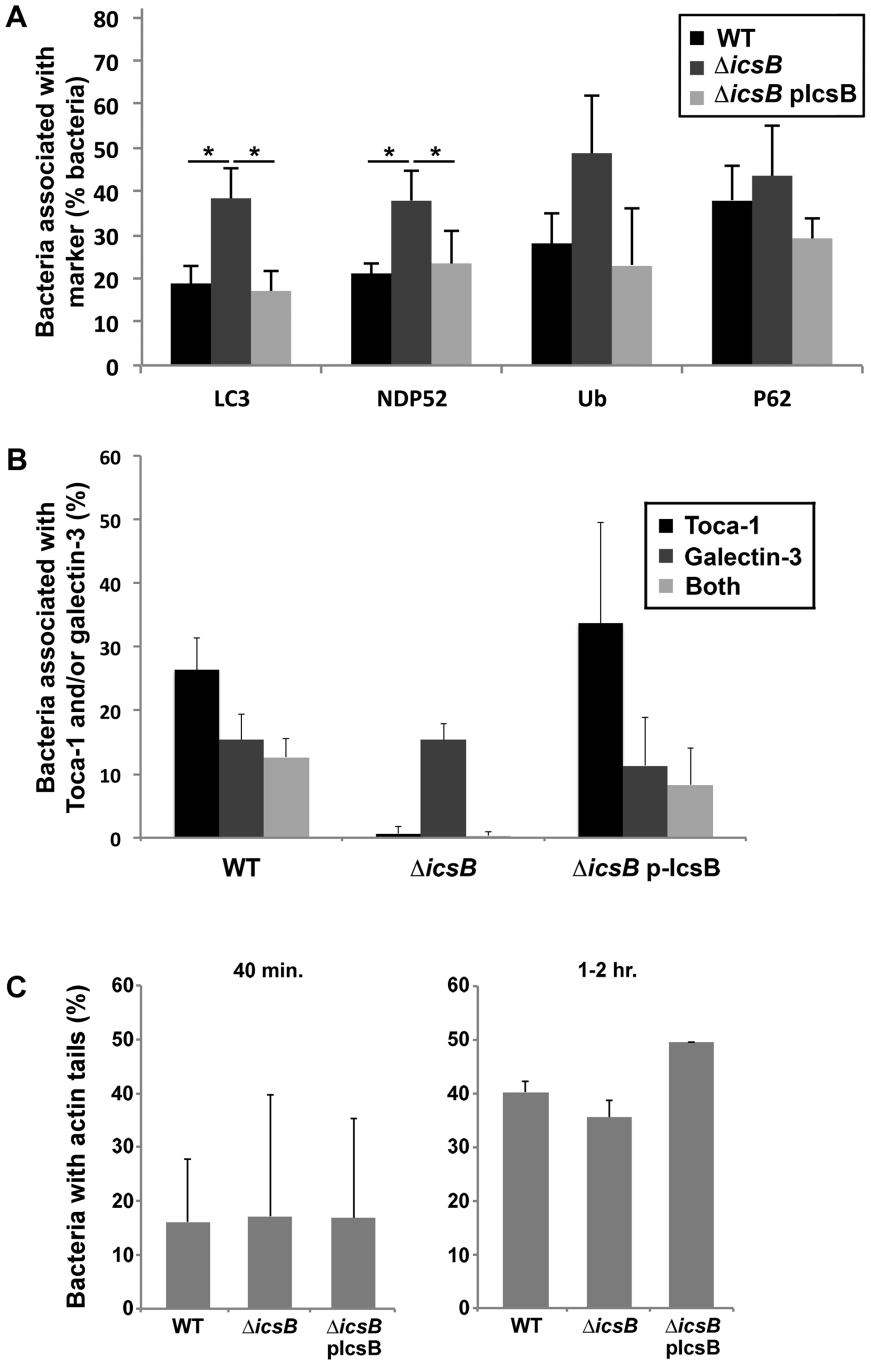
IcsB inhibits early recruitment of LC3 around *S. flexneri* that are cytoplasmic. (A) LC3, NDP52, ubiquitin and P62 recruitment in the vicinity of wild-type pflag (WT), Δ*icsB* pflag, or Δ*icsB* pIcsB-flag *S. flexneri*, at 40 min. of infection of HeLa cells that had been transfected with LC3-GFP or labeled with antibodies to other autophagy markers. *P<0.05. (B) Prevalence of galectin-3, Toca-1, or both around intracellular wild-type *S. flexneri* at 40 min. of infection. (C) Formation of actin tails at 40 min. (left graph) or 1-2 hr. (right graph) of infection by wild type (WT), Δ*icsB S. flexneri*, or Δ*icsB S. flexneri* complemented with pflag-IcsB. Data represent the mean ± S.D. of three independent experiments. Within each panel, the P value for each pair of data is >0.1. Data represent mean ± S.D. of three independent experiments.

To test whether IcsB altered the recruitment of these markers early in infection, we measured the prevalence of recruitment of LC3, NDP52, P62, and ubiquitin in the vicinity of intracellular *S. flexneri* in the presence or absence of IcsB at 40 min. post-invasion (Fig. 4A). In the absence of IcsB, recruitment of LC3 and NDP52 was significantly increased, and recruitment of P62 and ubiquitin was increased, albeit not to statistical significance. These data indicate that, soon after bacterial entry into host cells, IcsB can limit recruitment of these autophagy-related proteins to the vicinity of intracellular bacteria.

### Escape from the uptake vacuole and actin tail formation are independent of IcsB

In addition to being critical to the process of autophagy, components of the macroautophagy pathway participate in innate immune responses that are distinct from autophagy. Of particular interest here is LC3-associated phagocytosis, a process that occurs early upon uptake of pathogens and other cargo, involves several components of the classical autophagy pathway, and promotes phagosome maturation [16]–[18]. LC3-associated phagocytosis has been described for both phagocytic cells and non-phagocytic cells and is notably not associated with decreased pathogen survival [27]–[29]. Alternatively, LC3 has been observed at early times during *S. flexneri* infection associated with vacuolar membrane remnants [30], which similarly has not been shown to be associated with decreased pathogen survival. The presence of LC3 and NDP52 around a subset of intracellular *S. flexneri* at 40 min. of infection raised the possibility that LC3 recruitment was occurring in the process of LC3-mediated phagocytosis and/or associated with recognition of vacuolar membrane remnants.

In previous descriptions of LC3-associated phagocytosis, LC3 accumulates on phagosomal membranes. During *S. flexneri* infection of HeLa cells, bacteria lyse the phagosome beginning as early as 15 min. after bacterial contact with the cell, and essentially all phagosomes are lysed by 30 min. [31], whereas at 40 min. after contact, we observed LC3 around 15–20% of wild type and 35–40% of Δ*icsB* mutant bacteria (Fig. 4A). This raised the possibility that if LC3 was associated with the phagosomal membrane, the absence of IcsB caused a delay in lysis of the phagosome. To test this, we compared the recruitment of galectin-3 around the *icsB* mutant to that around the wild type strain at 40 min. of infection; since galectins bind β-galactosides on the luminal face of vesicles [32], galectin-3 serves as a marker of vacuolar lysis by *S. flexneri* [30], [33], [34]. At 40 min. of infection, galectin-3 was present around an equivalent percentage of Δ*icsB* mutant and wild type bacteria (Fig. 4B), suggesting that the absence of IcsB had no impact on phagosomal escape and that the differences in LC3 recruitment to the Δ*icsB* and the wild type strain were independent of phagosomal escape *per se*.

Since actin tails can only form after bacteria access the cytoplasm, the efficiency of actin tail formation can serve as an additional marker of access to the cytoplasm. Moreover, Toca-1 is required for efficient initiation of actin tail formation by intracellular *Shigella* [11]. Toca-1, which is recruited to intracellular bacteria prior to actin tail formation, relieves auto-inhibition of N-WASP, thereby activating N-WASP and enabling N-WASP-mediated actin polymerization. Based on this, we considered whether IcsB-mediated recruitment of Toca-1 to the bacteria might be required for initiation of actin tail formation. At 40 min. of infection, actin tail formation was similar for the Δ*icsB* mutant and the wild type strain (Fig. 4C, left graph). The same was also true at 1–2 hr. of infection (Fig. 4C, right graph). These results are consistent with the galectin-3 data indicating that phagosomal escape was similar for the Δ*icsB* mutant and the wild type strains. They also indicate that actin tail formation occurs independently of IcsB. Together with our previously published results [11], this observation demonstrates that for actin tails to form efficiently, Toca-1 must be present in the cell, but need not be enriched around bacteria. Thus, the relatively lower concentration of Toca-1 that is present diffusely in the cytosol is sufficient for efficient actin tail initiation.

### Early IcsB-dependent repression of LC3 recruitment is independent of IcsA

The IcsB-mediated inhibition of autophagy that occurs at later times during infection (4–6 hr) involves IcsB binding to the *Shigella* surface protein IcsA, which blocks IcsA recognition by the pro-autophagy factor Atg5 [15]. This pathway is dependent on IcsA, since at 4 hrs of infection, LC3 recruitment to an *icsA* mutant is minimal [15].

**Figure 5:**
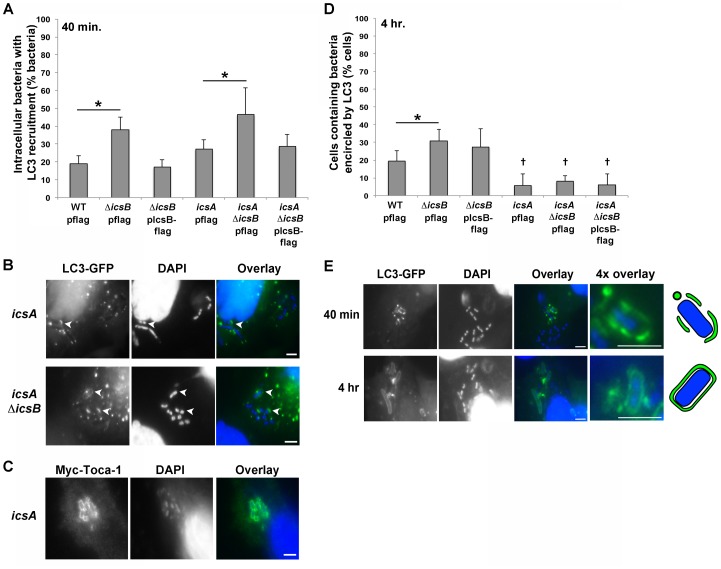
Early repression of LC3 recruitment is independent of IcsA. (A) LC3 recruitment to the vicinity of wild-type (WT), Δ*icsB*, or *icsA* Δ*icsB S. flexneri*, complemented or not with pIcsB-flag, at 40 min. of infection of HeLa cells that had been transfected with LC3-GFP. (B) Representative images corresponding to (A). Arrowheads, bacteria with LC3 recruitment. (C) Toca-1 recruitment to *icsA S. flexneri* at 40 min. of infection of HeLa cells that had been transfected with Myc-Toca-1, with immunofluorescence using antibody to Myc and staining with DAPI. (D) Cells containing LC3 encircling WT, Δ*icsB*, *icsA*, or *icsA* Δ*icsB S. flexneri*, complemented or not with pIcsB-flag, at 4 hr of infection of MEFs that had been transfected with LC3-GFP. (E) Distinctive LC3 distribution patterns around WT *S. flexneri* at early (40 min., top panels) and late (4 hr., bottom panels) times after infection. Data represent the mean ± S.D. of three independent experiments. *P<0.05. Images are representative. †, each bar was statistically different from bars for isogenic strain that contains *icsA*. Scale bars, 5 µm.

To test whether early IcsB-dependent repression of LC3 recruitment was dependent on IcsA, we compared LC3 recruitment in the vicinity of strains containing or lacking IcsA at early and late infection times. At 40 min., whether or not IcsA was present, the prevalence of LC3 recruitment significantly increased in the absence of IcsB (Fig. 5A and B), indicating that this early process is independent of IcsA. Moreover, Toca-1 recruitment was independent of *icsA* (Fig. 5C). In contrast, at 4 hrs of infection, levels of autophagy were significantly lower in each strain that lacked IcsA compared to the IcsA+ strains (Fig. 5D; †, P<0.05), confirming the previous observation that at late times, an IcsA-dependent autophagy inhibition mechanism predominates [15]. Thus, *Shigella* IcsB interferes with two distinct processes, an early one that is IcsA-independent, and a late autophagy pathway that is IcsA-dependent.

When present, the pattern of LC3 recruitment was notably distinct at the two times we analyzed (Fig. 5E and data not shown). At early times (40 min.), LC3 appeared fragmented with punctate foci adjacent to bacteria, while at late times (4 hr.), LC3 appeared as a continuous signal that completely surrounded bacteria (Fig. 5E and data not shown). The pattern of LC3 observed at early times was reminiscent of that directed against membrane remnants [30], leading us to speculate that LC3 may be localized to membrane remnants as a result of either LC3-associated phagocytosis or autophagy triggered by membrane remnants. Indeed, galectin-3 and cholesterol, a membrane component, localized with Toca-1 in the vicinity of intracellular *S. flexneri* at this early infection time (Figs. 4B and S4).

### Relationship of Toca-1 to early LC3 recruitment

**Figure 6:**
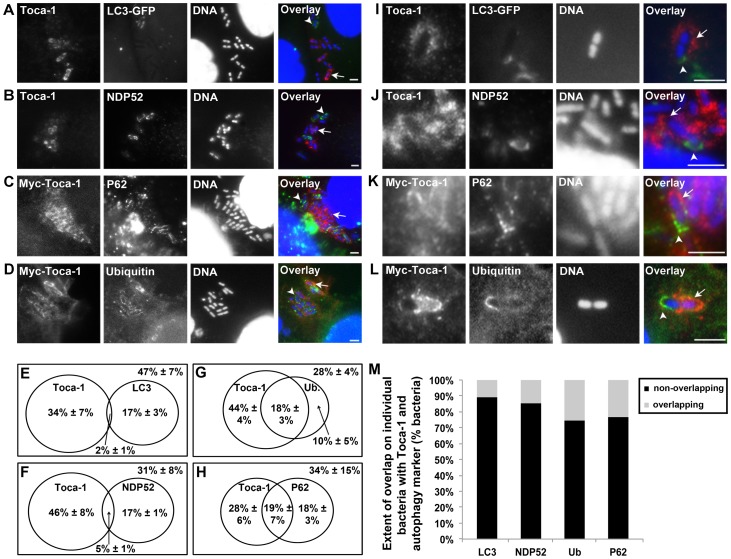
Toca-1 recruitment around wild-type bacteria is associated with decreased recruitment of LC3 and autophagy markers. **A–D**. Wild-type *S. flexneri* infection (40 min.) of HeLa cells that were untransfected or had been transfected with Myc-Toca-1 or LC3-GFP (A), with immunofluorescence using antibody to Toca-1 or Myc and to NDP52 (B), P62 (C) and ubiquitin (D). Arrowheads, bacteria exhibiting recruitment of autophagy-associated proteins. Arrows, bacteria exhibiting recruitment of Toca-1. (E–H) Venn diagrams of the distribution of Toca-1 and autophagy markers around individual intracellular bacteria for experiments shown in panels A-D. Numbers in the upper right corners of the rectangles represent the percentage of bacteria that were surrounded by neither marker. Data represent the mean ± S.D. of three independent experiments. (I–L) Magnified images of individual bacteria that were surrounded by both Toca-1 and LC3 (I), NDP52 (J), P62 (K), or ubiquitin (L). Distribution of Toca-1 (arrow) and autophagy marker (arrowhead) to distinct non-overlapping microdomains around each bacterium. (M) Quantification of overlap of signal around individual bacteria that exhibited recruitment of both Toca-1 and an autophagy protein. Data represent the mean of three independent experiments. Images are representative. Scale bars, 5 µm.

The requirement of IcsB for both Toca-1 recruitment and repression of LC3 recruitment raised the possibility that Toca-1 participated in limiting LC3 recruitment. Indeed, we found that the subpopulation of bacteria associated with Toca-1 was non-overlapping with the subpopulation associated with LC3. Two ±1% of intracellular bacteria were associated with both LC3 and Toca-1, compared with 17±3% associated with LC3 only and 32±7% with Toca-1 only (Fig. 6A and 6E).

Since Toca-1 recruitment anti-correlated with LC3 recruitment, we examined whether other autophagy-related factors were also anti-correlated with Toca-1 recruitment early during *S. flexneri* infection. We compared the subpopulation of bacteria associated with NDP52, p62, or ubiquitin to that associated with Toca-1. Very few intracellular bacteria were associated with both Toca-1 and NDP52 (5±1%; Fig. 6B and 6F). Moreover, in the cases of P62 and ubiquitin, whereas more than half of the bacteria associated with P62 or ubiquitin also displayed Toca-1 recruitment (Fig. 6C-D and 6G–H), a closer examination of individual bacteria exhibiting recruitment of both Toca-1 and an autophagy-associated protein revealed that the two proteins were restricted to distinct, non-overlapping microdomains around the bacteria (Fig. 6I–M). For more than 75% of the bacteria that were associated with both Toca-1 and another autophagy associated protein, the two signals were restricted to non-overlapping microdomains (Fig. 6M). Thus, even when Toca-1 and an autophagy-associated factor were recruited to the same bacterium, they were not co-localized.

### The pro-autophagy activity of Toca-1 observed during infection with *S*. Typhimurium is absent early during *S. flexneri* infection

**Figure 7:**
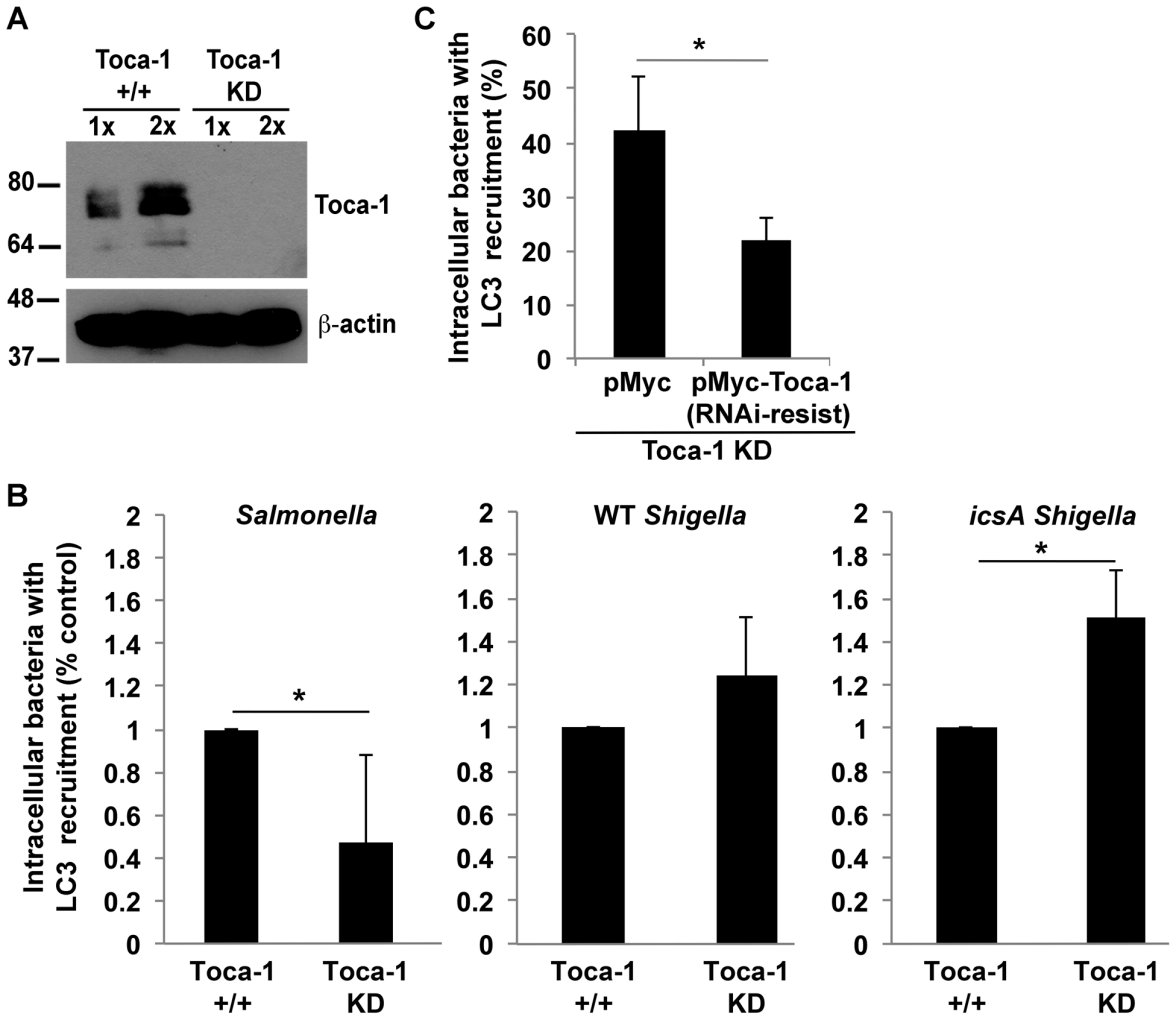
The pro-autophagy activity of Toca-1 observed during infection with *Salmonella* Typhimurium is absent in the setting of *S. flexneri* infection. (A) Levels of Toca-1 in stable Toca-1 KD or control (Toca-1^+/+^) cells by western blot. 1x and 2x, relative loading. (B) LC3 recruitment around *S*. Typhimurium or WT or *icsA S. flexneri* at 40 min. of infection of Toca-1^+/+^ or Toca-1 KD cells. For each strain, the values are normalized to LC3 recruitment in the presence of Toca-1. (C) LC3 recruitment around *icsA S. flexneri* at 40 min. of infection of Toca-1 KD cells complemented with Myc-tagged shRNA-resistant Toca-1 [pMyc-Toca-1 (RNAi-resist)] or Myc alone. Data represent the mean ± S.D. of three independent experiments. *P<0.05.

These findings suggested that the function of Toca-1 might be very different in the setting of *S. flexneri* infection from its role in supporting autophagy of intracellular *S*. Typhimurium. During *S*. Typhimurium infection, Toca-1 co-localizes with LC3 around a subset of intracellular bacteria and promotes anti-bacterial autophagy [35]. We postulated that in the context of *S. flexneri* infection, rather than functioning to promote LC3 recruitment, Toca-1 was either neutral or inhibitory in this function. We tested the role of Toca-1 in LC3 recruitment by comparing LC3 association with bacteria at 40 min. of infection in the presence or absence of Toca-1 (Fig. 7A), using cells stably transfected with a short hairpin RNA (shRNA) directed against Toca-1 or, as a control, an empty lentiviral vector. During *S*. Typhimurium infection, the absence of Toca-1 was associated with a significant decrease in LC3 recruitment (Fig. 7B, left graph), as described previously [35]. So as to control for any effect actin tail formation might have on this process, in addition to examining LC3 recruitment around wild type *S. flexneri*, we examined its recruitment around the *icsA* mutant. The absence of Toca-1 was associated with an increase in LC3 recruitment that reached significance for the *icsA* mutant and was a trend for the wild-type strain (Fig. 7B, right and middle graphs, respectively). Complementation of the Toca-1 depletion using an shRNA-resistant form of Toca-1 rescued repression of LC3 recruitment by the *icsA* mutant (Fig. 7C), indicating that the increase in LC3 recruitment observed was due to the absence of Toca-1 *per se*. These data indicate that in the context of *Shigella* infection, Toca-1 does not promote LC3 recruitment, but rather is neutral in this respect, and in the absence of *Shigella* actin tail formation, represses it.

**Figure 8:**
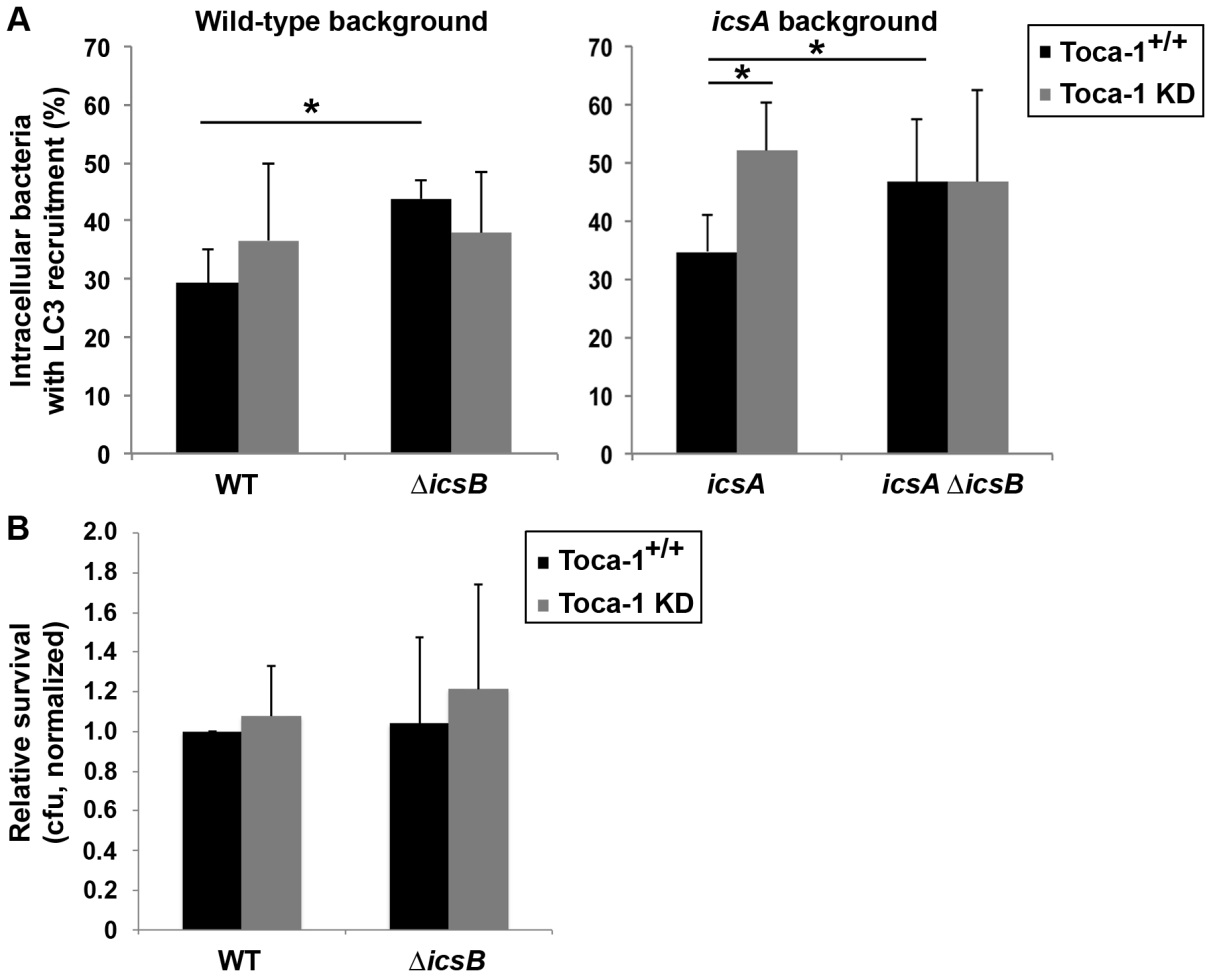
IcsB mediates repression of LC3 recruitment and increased survival independent of IcsA in the presence of Toca-1. (A) LC3 recruitment around wild-type (WT) versus Δ*icsB* (left graph) or around *icsA* versus *icsA* Δ*icsB* (right graph) *S. flexneri* at 40 min. of infection of Toca-1^+/+^ or Toca-1 KD cells. (B) Survival of wild type or Δ*icsB S. flexneri* at 2.5 hrs. of infection by gentamicin protection assay in Toca-1^+/+^ or Toca-1 KD cells. Data represent the mean ± S.D. of three independent experiments. *P<0.05.

We demonstrated above that early during infection, IcsB is required both for Toca-1 recruitment (Figs. 1 and S1) and for repression of LC3 recruitment (Fig. 4A–B) around intracellular *S. flexneri*. To test whether the role of IcsB in limiting LC3 recruitment depends on Toca-1, we compared LC3 recruitment around strains expressing or lacking IcsB in cells containing or lacking Toca-1. In both the wild type and the *icsA* mutant background, IcsB played a role in limiting LC3 recruitment only in the presence of Toca-1 (Fig. 8A).

As in Fig. 7B, the increase in LC3 recruitment that occurred in the absence of Toca-1 reached statistical significance for the *icsA* mutant and was a trend for the wild-type strain (Fig. 8). The differences observed for the wild type and the *icsA* mutant may be a result of functional overlap between actin tail formation and repression of LC3 recruitment around stationary bacteria. We postulate that in the presence of IcsA, bacteria can escape LC3 recruitment either by forming actin tails, which would enable them to move away from LC3-studded membrane fragments, or via IcsB-mediated repression of LC3; wild type bacteria are able to form actin tails independent of whether they secrete sufficient IcsB to repress LC3 recruitment. For the *icsA* mutant, actin tail formation does not occur, so that IcsB-mediated repression of LC3 is the only option for escape from being marked with LC3; thus, the *icsA* mutant serves as a tool for evaluating IcsB-mediated repression of LC3 in relative isolation. Consistent with this possibility, under each condition, the percentage of bacteria associated with LC3 trended higher for the *icsA* mutant than for the wild-type strain (Fig. 8A).

It seemed unlikely that LC3 recruitment to intracellular *S. flexneri* early during infection was associated with an autophagy pathway targeting bacterial survival, since the absence of IcsB led to significant increases in LC3 recruitment (Fig. 4A), yet the absence of IcsB has no effect on bacterial survival up to 3 hr of infection [15]. To further explore whether LC3 recruitment to *S. flexneri* at these early times might be associated with autophagy, we determined the impact of Toca-1-associated repression of LC3 recruitment on *S. flexneri* survival. At 2–1/2 hr of infection, we found no difference in bacterial survival in the Toca-1 KD versus Toca-1^+/+^ cells for either the wild type or the Δ*icsB* strain (Fig. 8B), indicating that absence of Toca-1 does not alter *Shigella* survival and lending support to the possibility that LC3 recruitment early during infection may be indicative of either LC3-associated phagocytosis or an autophagy pathway directed against membrane remnants that does not target bacteria directly for destruction.

## Discussion

The host protein LC3 is best described for the role of the lipidated form in the formation of autophagosomes during autophagy. However, a distinct yet critical role for lipidated LC3 is in LC3-associated phagocytosis, wherein it marks phagosomal membranes in a process that is independent of autophagy [16]. We describe LC3 recruitment around a subset of *S. flexneri* soon after entry into MEFs and other non-phagocytic mammalian cells. LC3 recruitment around *S. flexneri* at this time appears to be unrelated to autophagy targeting bacteria for destruction, as conditions that lead to significant changes in the level of LC3 recruitment did not lead to reductions in bacterial survival (Figs. 4A and 8, and [15]). Moreover, *Shigella* IcsB, which we show represses LC3 recruitment (Fig. 1), does not contribute to inhibition of autophagy-related killing of *S. flexneri* at these early infection times [15]. Given evidence that recruitment of LC3 around *S. flexneri* at 40 min. of infection is not promoting bacterial killing, we conclude that it likely represents either LC3-associated phagocytosis or activation of autophagy targeting membrane remnants that serves a purpose other than destruction of intracellular bacteria.

LC3-associated phagocytosis is a poorly understood process that is characterized by LC3 studding of membranes of intact phagosomes [16], [17]. At least in some cases, the process promotes maturation of the phagosome [16]–[18]. Several features described in this manuscript suggest that the process we observe is distinct from classical descriptions of LC3-associated phagocytosis in that, at the time we characterize (40 min. after bacterial contact with the cell), the bacterial cargo is no longer within intact phagosomes, but rather that LC3 is associated with phagosomal membrane fragments that surround cytoplasmic bacteria. First, recruitment of Toca-1 is initiated during bacterial uptake into cells (Fig. S2), concurrent with the formation of membrane ruffles that will constitute phagosomes that engulf entering bacteria. Within minutes of uptake, *Shigella* lyse the phagosome, and phagosomal membrane remnants surround intracellular *S. flexneri* at early infection times [30], [34], [36]–[38]. It seems likely that Toca-1 and IcsB remain associated with the phagosomal remnants after uptake and phagosomal lysis has occurred. Secondly, galectin-3, a marker of damaged or lysed vacuolar membranes, and cholesterol, a component of the membrane, are enriched in the vicinity of bacteria associated with Toca-1 (Figs. 4B and S4). And thirdly, at this early infection time, LC3 is typically distributed in fragments adjacent to and partially surrounding bacteria (Fig. 5E), a pattern strikingly similar to that observed for membrane remnants early after *S. flexneri* entry [30], [34]. This suggests that assembly of LC3-associated machinery occurs during the process of cargo engulfment. These findings also suggest that LC3-mediated phagocytosis does not impede *Shigella* lysis of the phagosomal membrane and that the LC3-associated machinery does not immediately dissociate from the phagosomal membrane upon its fragmentation. Whether the LC3-associated phagocytosis machinery contributes to recycling of phagosomal membrane fragments during *S. flexneri* infection, as suggested by other studies [30], will require additional investigation.

Several cellular factors have been shown to be required for LC3-associated phagocytosis, including Atg5, Atg7, and Beclin-1, PI3 kinase activity and the generation of reactive oxygen species [16]–[18], [27], [28]. In distinct cellular circumstances, each of these also participates in autophagy, highlighting the overlap of LC3-associated phagocytosis with autophagy pathways. Dupont et al. [30] have described LC3 recruitment to vacuolar membrane remnants around a subset of intracellular *S. flexneri* early during infection. LC3 recruitment in this case may be promoting autophagy of membrane remnants in a manner that is independent of autophagy of the bacteria *per se*. Indeed, previous descriptions of recruitment of autophagy markers to vacuolar membrane remnants during *Shigella* infection have not demonstrated a role for autophagy marker recruitment in bacterial survival *per se* [23], [30], [34]. In the case of *Shigella* infection, since membrane remnants are derived from the phagocytic vacuole, the recruitment of LC3 to these membrane remnants may be the same process LC3-associated phagocytosis.

We found that LC3 recruitment to intracellular *S. flexneri* is inhibited by IcsB (Fig. 1). LC3-associated phagocytosis has been described for the intracellular pathogen *Burkholderia pseudomallei* [39]. Of note, the *B. pseudomallei* type three secreted effector BopA, a homolog of IcsB, inhibits LC3 recruitment to *B. pseudomallei* phagosomes [39]. This lends further support to our conclusion that the process described herein represents IcsB inhibition of an LC3-associated phagocytosis-like process and provides support to the idea that, during infection by *Shigella*, *Burkholderia*, and possibly other pathogens, LC3-associated phagocytosis may be the same process as LC3 recognition of vacuolar membrane remnants. A notable difference in the function of these two proteins is that whereas BopA contributes to lysis of the phagosome [39], we found no evidence that IcsB altered phagosome lysis (Fig. 4B–C).

Our findings together with these published results identify IcsB and BopA as a conserved family of type three effectors proteins that inhibit recruitment of LC3 to pathogen-associated phagosomal structures. In addition, they identify IcsB is a bacterial factor that modulates early recruitment of LC3 to *Shigella*. BopA and IcsB are the only bacterial factors reported thus far as inhibitors of this process.

Travassos et al. [23] examined LC3 recruitment around intracellular *S. flexneri* at slightly later infection times. At that time, LC3 was present around a subset of intracellular bacteria, and the number of bacteria associated with LC3 was increased for an *icsB* mutant, suggesting that for at least some bacteria, the association of bacteria with LC3 that we observed persists. LC3 recruitment was defective in the absence of the pattern-recognition molecule Nod1. In addition, the autophagy protein Atg16 was recruited to bacterial entry sites, dependent on the Nod1 and Nod2 [23]. An effect of Nod1 on survival of *S. flexneri* was documented only at 6 hr of infection, raising the possibility that the events described by these authors early during infection are part of the same process we describe here.

Previous work has shown that at later times during *S. flexneri* infection (4–5 hr), IcsB inhibits anti-bacterial autophagy by blocking Atg5 recognition of IcsA at the bacterial surface [15], [40]. Of note, whereas IcsB inhibition of autophagy depends on IcsA [15], we found here that IcsB inhibition of LC3 recruitment is independent of IcsA (Fig. 5A). Moreover, the absence of IcsA had no effect on the recruitment and localization of Toca-1 or IcsB. Together, these results suggest that IcsB inhibits innate immune responses in two distinct ways, first, by inhibiting LC3-associated phagocytosis and/or LC3 recruitment to vacuolar membrane fragments early during infection, and second, by inhibiting autophagy late during infection.

We observed that at early infection times (40 min.), IcsB recruited the host protein Toca-1 to intracellular bacteria (Fig. 1). This raised the possibility that Toca-1 would be required for inhibition of LC3 recruitment. Since Toca-1 is required for efficient formation of actin tails by *S. flexneri* [11], and actin tail formation likely enhances bacterial movement away from phagosomal membrane remnants, to test the role of Toca-1 in early LC3 recruitment *per se*, we examined LC3 recruitment for *icsA* mutant bacteria, which are unable to form actin tails [41], [42]. We found that Toca-1, like IcsB (Fig. 5A), is required for inhibition of LC3 recruitment around intracellular *S. flexneri* (Fig. 7B, right panel), thus identifying Toca-1 as a protein that modulates early LC3 recruitment.

Toca-1 is an F-BAR domain containing protein implicated in integration of regulation of the actin cytoskeleton with membrane trafficking [12], [43]–[45]. F-BAR domains are membrane-binding scaffolds that typically sense and promote positive curvature membrane deformation that leads to inward invagination of the membrane [46]. Toca-1 functions both in activation of the actin nucleation-promoting factor N-WASP and in autophagy [12], [35]. Indeed, we have shown previously that Toca-1 is required for efficient initiation of actin tail formation by *S. flexneri* [11]. Our finding here that Toca-1 is required for inhibition of LC3 recruitment identifies it as a cellular factor that likely modulates LC3-associated phagocytosis and/or LC3 recognition of membrane fragments. Moreover, the observation that Toca-1 recruitment is mutually exclusive with ubiquitination and recruitment of autophagy adaptor proteins raises the possibility that Toca-1 may be interfering with an early step in pathogen recognition. Our findings add Toca-1 to the list of factors that likely function both in LC3-associated phagocytosis and in autophagy. Under the conditions of our investigations described herein, Toca-1 appears to inhibit, rather than promote, LC3-associated phagocytosis and/or LC3 recognition of membrane fragments. Moreover, the contrasting function of Toca-1 during infection with *S*. Typhimurium [35] raises the possibility that Toca-1 plays opposing functions at different stages of bacterial infection and/or in the context of different pathogens.

## Supporting Information

Figure S1 **Recruitment of Toca-1 to intracellular bacteria lacking individual type three-secreted effector proteins.** Infection for 40 min. of HeLa cells that had been transfected with Myc-Toca-1 by a panel of 21 isogenic *S. flexneri* strains, each harboring a deletion of a single gene encoding a type three effector protein, followed by immunofluorescence using antibody to Myc. Images are representative. Arrowheads, bacteria with Toca-1 recruitment.
(TIF)

Figure S2 **Localization of Toca-1 to membrane ruffles.** Localization of Toca-1 around bacteria in membrane ruffles during entry (40 min. after contact) of wild-type *S. flexneri* into HeLa cells that had been transfected with Myc-Toca-1. Immunofluorescence using antibody to Myc, phalloidin staining of polymerized actin, and DAPI staining of DNA. Images are representative. Size bar, 5 µm.
(TIF)

Figure S3 **Recruitment of Toca-1 truncation mutants to intracellular** ***S. flexneri*** **.** *S. flexneri* infection for 40 min. of HeLa cells transfected with full length Myc-Toca-1 or one of the ten Myc-Toca-1 truncation mutants, followed by immunofluorescence with antibody to Myc and staining with phalloidin (polymerized actin) and DAPI (DNA). The residues included in each truncation mutant are indicated to the left. Scale bars, 5 µM.
(TIF)

Figure S4 **Localization of galectin-3 and cholesterol around** ***S. flexneri*** **early in infection.** Wild-type *S. flexneri* infection (40 min) of HeLa cells that had been transfected with Myc-Toca-1, followed by labeling of galectin-3 and Myc by immunofluorescence and staining of cholesterol with filipin and of DNA with DAPI. In overlays, Myc-Toca-1 is green, cholesterol and DAPI are blue, and galectin-3 is red. Size bars, 5 µm. Images are representative.
(TIF)
