# Supplementary figures and images for: Host and Bacterial Proteins That Repress Recruitment of LC3 to Shigella Early during Infection
Source: PLoS One. 2014 Apr 10;9(4):e94653. doi: 10.1371/journal.pone.0094653 (PMC3983221; doi:10.1371/journal.pone.0094653)

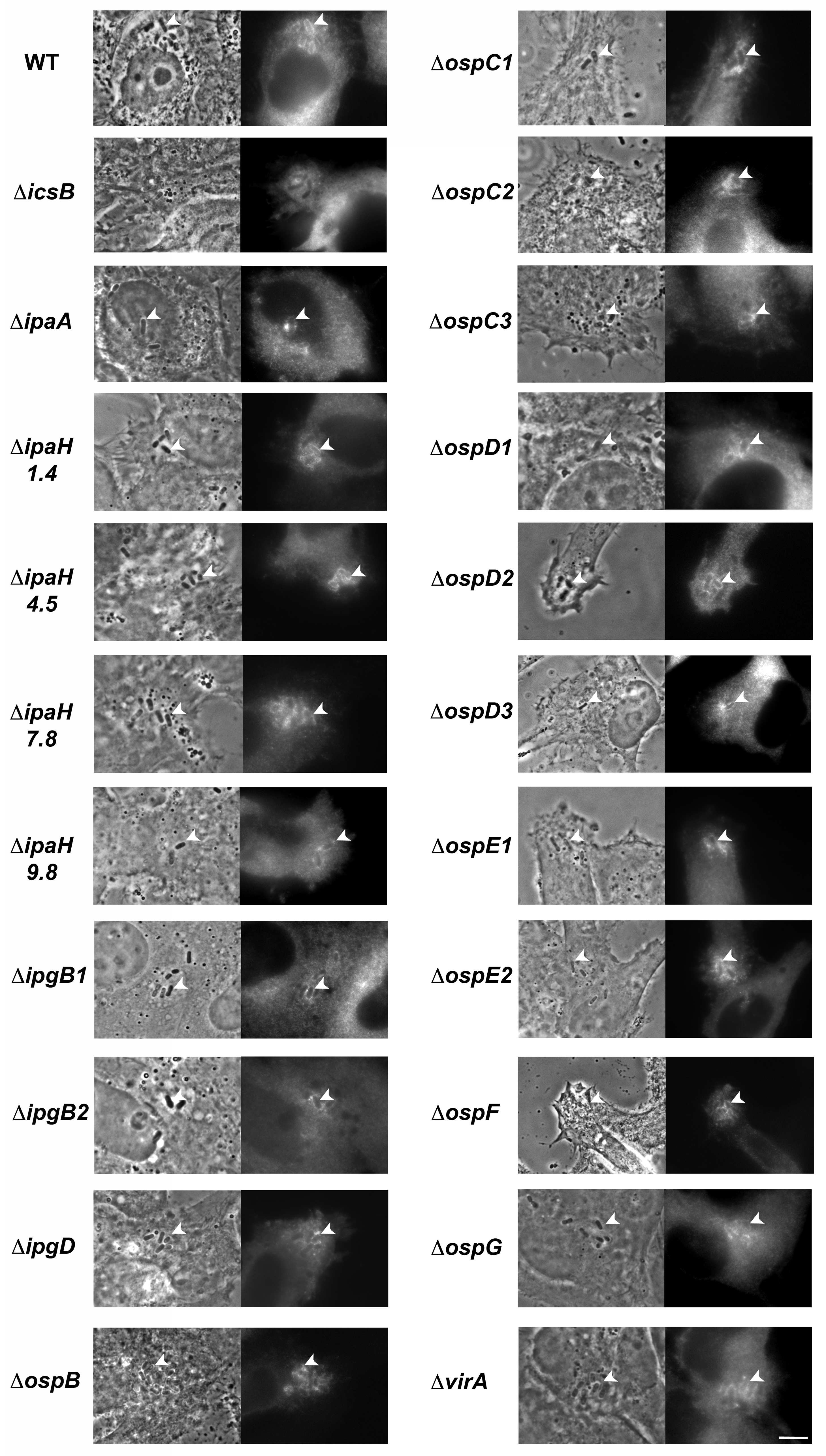

Supplement: Figure S1 — Recruitment of Toca-1 to intracellular bacteria lacking individual type three-secreted effector proteins. Infection for 40 min. of HeLa cells that had been transfected with Myc-Toca-1 by a panel of 21 isogenic S. flexneri strains, each harboring a deletion of a single gene encoding a type three effector protein, followed by immunofluorescence using antibody to Myc. Images are representative. Arrowheads, bacteria with Toca-1 recruitment. (TIF) [file pone.0094653.s001.tif]

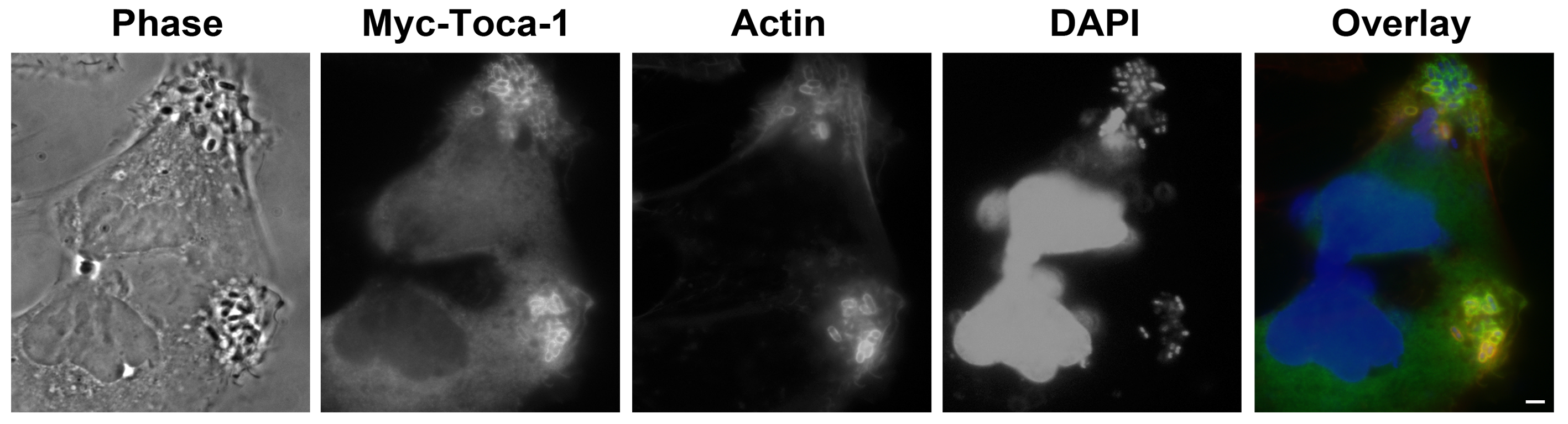

Supplement: Figure S2 — Localization of Toca-1 to membrane ruffles. Localization of Toca-1 around bacteria in membrane ruffles during entry (40 min. after contact) of wild-type S. flexneri into HeLa cells that had been transfected with Myc-Toca-1. Immunofluorescence using antibody to Myc, phalloidin staining of polymerized actin, and DAPI staining of DNA. Images are representative. Size bar, 5 µm. (TIF) [file pone.0094653.s002.tif]

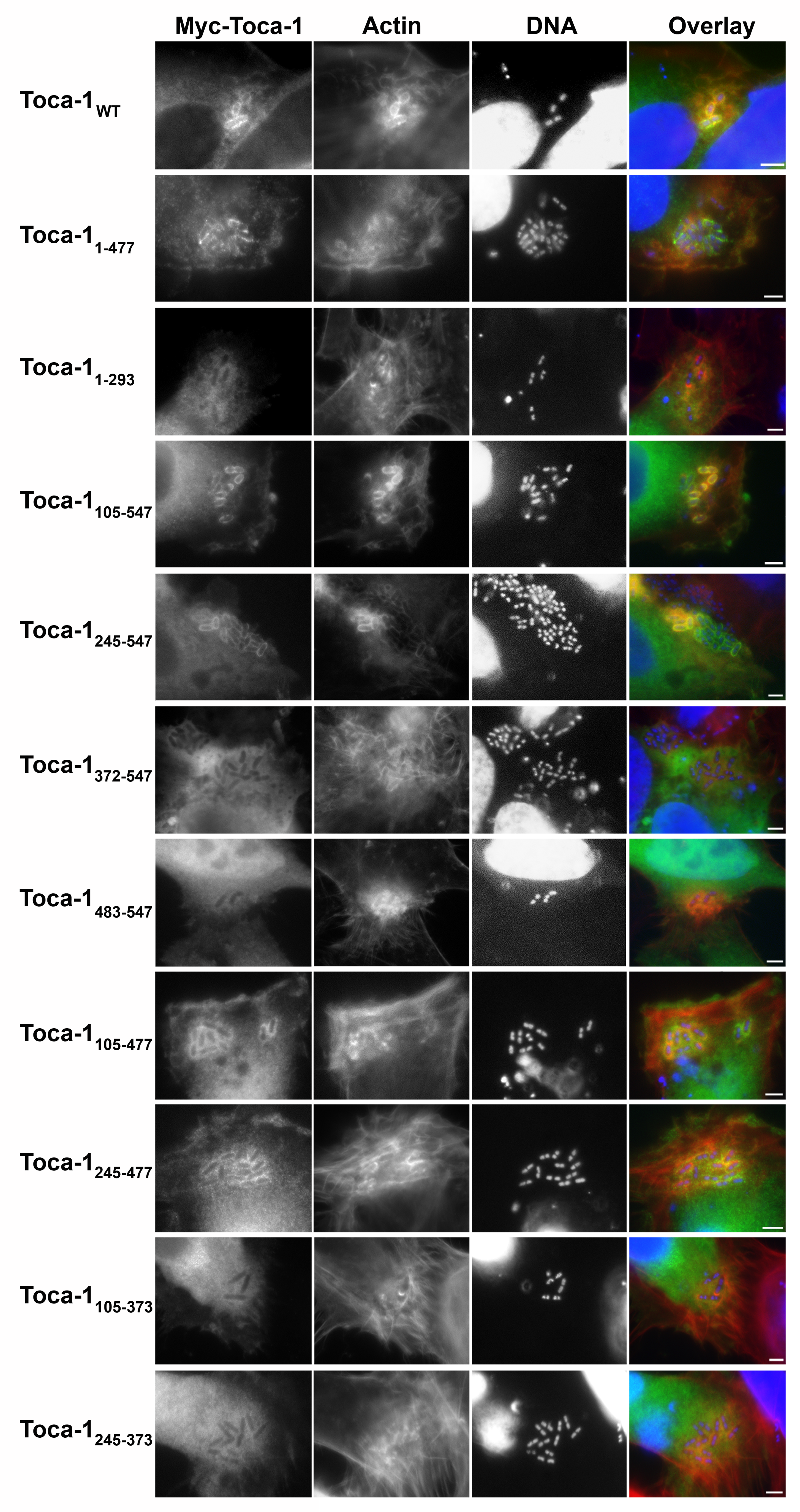

Supplement: Figure S3 — Recruitment of Toca-1 truncation mutants to intracellular S. flexneri . S. flexneri infection for 40 min. of HeLa cells transfected with full length Myc-Toca-1 or one of the ten Myc-Toca-1 truncation mutants, followed by immunofluorescence with antibody to Myc and staining with phalloidin (polymerized actin) and DAPI (DNA). The residues included in each truncation mutant are indicated to the left. Scale bars, 5 µM. (TIF) [file pone.0094653.s003.tif]

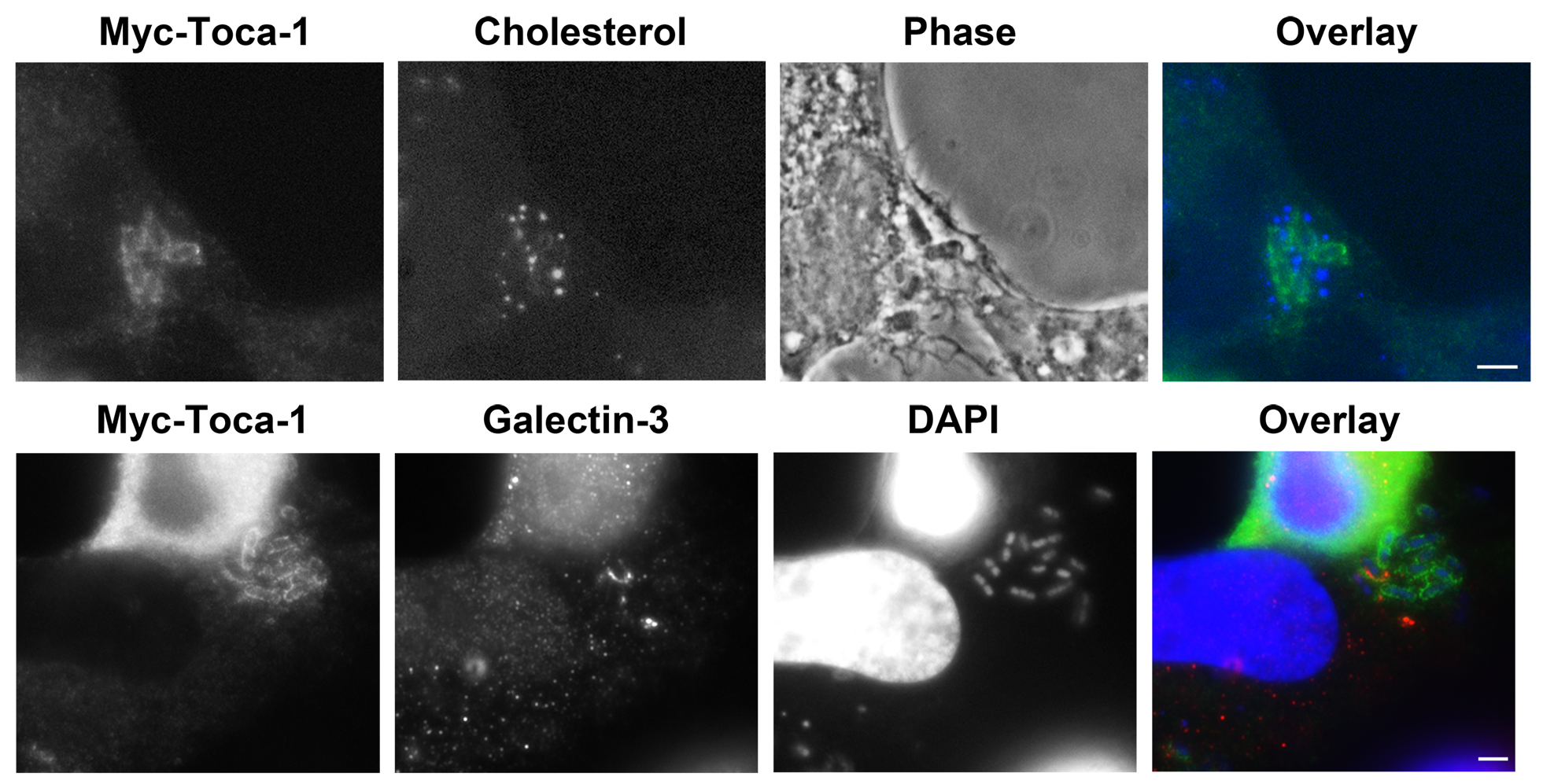

Supplement: Figure S4 — Localization of galectin-3 and cholesterol around S. flexneri early in infection. Wild-type S. flexneri infection (40 min) of HeLa cells that had been transfected with Myc-Toca-1, followed by labeling of galectin-3 and Myc by immunofluorescence and staining of cholesterol with filipin and of DNA with DAPI. In overlays, Myc-Toca-1 is green, cholesterol and DAPI are blue, and galectin-3 is red. Size bars, 5 µm. Images are representative. (TIF) [file pone.0094653.s004.tif]
